# Bioinformatic Analysis Identifying PSMB 1/2/3/4/6/8/9/10 as Prognostic Indicators in Clear Cell Renal Cell Carcinoma

**DOI:** 10.7150/ijms.71152

**Published:** 2022-05-01

**Authors:** Jing-Yi Guo, Zuo-qian Jing, Xue-jie Li, Li-yuan Liu

**Affiliations:** 1 Department of Urology, The First Affiliated Hospital of China Medical University, Shenyang 110001, China.; 2 Department of Ophthalmology, The First Affiliated Hospital of China Medical University, Shenyang 110001, China.

**Keywords:** clear cell renal cell carcinoma, PSMB, bioinformatic analysis, prognosis

## Abstract

Renal cancer incidence has been increasing across the world, clear cell renal cell carcinoma (ccRCC) represents the major subtype of renal cancer. The proteasome is involved in onset, metabolism and survival of tumor and has been recognized as a therapeutic target for various malignancies, while the role of β subunits of proteasome, PSMB gene family, in ccRCC has not been fully unveiled. Herein we investigated the expression and the prognostic role of PSMBs in ccRCC by analyzing a series of databases, including ONCOMINE, UALCAN, cBioPortal, STRING, GEPIA, GO and KEGG. Over-expressions of PSMB1/2/4/7/8/9/10 mRNA were found in ccRCC tissues compared to normal tissues, transcriptional levels of PSMB2/3/4/6/8/9/10 were significantly positively associated with patients' individual cancer stages and grades. Similar or higher levels of proteins encoded by PSMB1/2/3/7/8/9/10 were observed in tumor tissues compared to normal renal tissues. Further, high mRNA levels of PSMB1/2/3/4/6/10 were correlated with shorter overall survival in univariate analysis. Taken together, the results of our analysis implied that overexpression of PSMB1/2/3/4/6/8/9/10 were indicative of worse prognosis of ccRCC. However, further researches were required to validate our findings.

## Introduction

Renal cell carcinoma (RCC) originates from the renal tubular epithelium and makes up more than 90% of cancers in the kidney 1. Worldwide, RCC represents the sixth most frequently diagnosed cancer among males and the tenth among females, resulting in 5% and 3% of all cancer diagnoses, respectively 2. Clear cell renal cell carcinoma (ccRCC) is the most common histological subtype of RCC, accounting for approximately 75% of cases and the majority of metastasis as well as kidney cancer deaths 1, 3. Localized RCC are best treated with surgery whereas metastatic RCC is refractory to conventional chemotherapy 1. Moreover, despite nephrectomy with curative intent, 30% of patients diagnosed with localized ccRCC eventually develop metastases 4-7. Though smaller renal tumors are being detected with the help of more sensitive abdominal imaging, locally advanced cases continues to be diagnosed in a considerable proportion of patients 8. According to the latest statistics provided by the World Health Organization, RCC was ranked the 13th most lethal carcinoma worldwide 8. Therefore, efforts have been made in improving the prognosis of the diagnosed patients. For instance, The Cancer Genome Atlas (TCGA) denoted the somatic genetic and genomic alterations in RCC, biomarkers of poor prognosis were also identified 3. Targeted agents such as sorafenib, sunitinib, bevacizumab which inhibit vascular endothelial growth factor and its receptor and everolimus and temsirolimus which inhibit mTOR complex I were approved clinically 1. However, ccRCC is a highly genetically heterogeneous disease, in a study of four patients with ccRCC who had multiple tumors were subjected to multi-region genetic analysis, common driver events such as SETD2 , PBRM1 , MTOR , PIK3CA, PTEN and KDM5C mutations were observed heterogeneously within the primary tumour and metastatic sites - in some regions but not others 9. Therefore, there is good reason to suppose distinct effective therapies and prognosis for different individuals. However, a majority of molecules that have potential prognostic values remain unexplored. PSMBs gene family might be a good candidate.

PSMBs gene family encodes β subunits of the proteasome which is in charge of post- ubiquitination proteasomal degradation of aberrantly folded or typically short-lived intracellular proteins. The proteasome is the central player of the ubiquitin-proteasome system (UPS) which degrades more than 80% of intracellular proteins 10. Dysregulation of UPS is closely related to tumor, cancer cells have been found to utilize the UPS to achieve aberrant proliferation and resistance to apoptosis as well as degradation of tumor suppressive proteins that would otherwise impede their growth and division 10, 11. For instance, the VHL tumor suppressor gene is inactivated in greater than 60% of RCC cases. The VHL protein (pVHL) serves as an ubiquitin activating enzyme (E3 ligase) that targets HIF-1, the hypoxia inducible transcription factor, inactivated pVHL thus leads to aberrant upregulation of HIF-1 and hypervascularity of renal tumors 12. Besides, proteasome inhibitors bortezomib and carfilzomib have been observed to cause apoptosis of kidney cancer cell lines 13-16.

The proteasome is a multicatalytic proteinase complex with a highly ordered ring-shaped 20S core structure. The core structure is composed of 4 rings of 28 non-identical subunits; 2 rings are composed of 7 α subunits and another 2 rings are composed of 7 β subunits 17. β-rings form a proteolytic chamber and α-rings serve as a gate for entry into the chamber. Of these 14 subunits, β1, β2 and β5 subunits (encoded by PSMB6, PSMB7, and PSMB5 respectively) carry out the hydrolysis as Thr proteases for the cleavage of peptide bonds at the carboxyl-terminal side after acidic, basic and hydrophobic residues, respectively 18. To date, 11 PSMBs have been identified in human genomes and numbered in the order of their discovery (PSMB1, PSMB2, PSMB3, PSMB4, PSMB5, PSMB6, PSMB7, PSMB8, PSMB9, PSMB10, PSMB11). Four non-catalytic subunits β3, β4, β6 and β7 are encoded by PSMB3, PSMB2, PSMB1 and PSMB4, respectively. By the replacement of the subunits β1, β2 and β5 with alternate catalytic subunits β1i, β2i and β5i (encoded by PSMB9, PSMB10, and PSMB8 respectively) which is induced by interferon (IFN), another subtype which is named the immunoproteasome is formed 19. The subunit β5t, which is encoded by PSMB11, is expressed in approximately 80% of human thymic cortex. By replacing subunits β1, β2 and β5 with subunits β1i, β2i and β5t, thymoproteasomes are formed 20.

Increased aberrant expressions of PSMBs have been reported in several human malignancies, some might act as molecular therapeutic target 21, 22. However, expressions of the majority of the PSMBs gene family in renal cancer have not been sufficiently elucidated. Due to the complexity of various subunits' functions and potential interrelationships among different subunits 23, 24, it is helpful to summarize the expressions of PSMB family members in RCC patients. In the present study, we conducted comprehensive analysis on the expression and prognostic value of genes PSMB1-10 in ccRCC based on a series of large databases, the newly found gene PSMB11 was not included in the analysis due to the absence of data in the majority of database.

## Materials and methods

### Oncomine database

Oncomine database (www.oncomine.org) is a cancer microarray database and a web-based data-mining platform aimed at facilitating discovery from genome-wide expression analyses 25. In this study, mRNA levels of PSMB1-10 of renal cancer tissues and their corresponding adjacent normal control tissues were obtained from ONCOMINE database. Student's t-test was used to compare the differences between transcriptional expressions of cancer tissues and normal samples. Cut-off of p value and fold change were as following: p value: 0.01, gene rank: 10%, data type: mRNA.

### UALCAN

UALCAN is an interactive web-portal (http://ualcan.path.uab.edu) to perform in-depth analyses of TCGA gene expression data. It uses level 3 RNA-seq and clinical data of 31 cancer types to perform analysis, allowing users to analyze relative expression of genes of interest across tumor and normal samples as well as in various tumor subgroups based on individual cancer stages, tumor grade or other clinicopathologic parameters 26. In this study, UALCAN was used to analyze the mRNA expressions of PSMB1-10 in subgroups of ccRCC tissues and their adjacent normal renal tissues. Difference in transcriptional expressions was compared by Student's t test and p <0.01 was considered as statically significant.

### Human Protein Atlas

The Human Protein Atlas (HPA) (https://www.proteinatlas.org) is an information database of protein expression patterns in normal human tissues, in cells, and in cancer for nearly 20 kinds of cancers 27. Users can identify tumor-type specific proteins expression patterns in a given type of cancer. The single cell RNA sequencing (scRNA seq) dataset of the HPA website is based on meta-analysis of literature on scRNA seq and single cell databases that include healthy human tissue. The total read counts for all genes in each cell cluster was calculated by adding up the read counts of each gene in all cells belonging to the corresponding cluster. And the read counts were normalized to transcripts per million (nTPM) protein coding genes for each of the single cell clusters. Z-score is when you normalize a variable such that the standard deviation is 1 and the mean is 0. Thus, all the genes are easier to compare, as they have the same center and distribution. In this study, immunohistochemistry images of proteins encoded by PSMB1-10 in the glomeruli and tubules of human normal tissues and ccRCC tissues were obtained from the website to perform a comparison. The heatmap shows expression of PSMB1-10 in different single cell type clusters of the kidney.

### TCGA database

TCGA database is a public funded project that aims to provide publicly available datasets to help improve diagnostic methods, treatment standards, and finally to prevent cancer. Large-scale genome sequencing and integrated multi-dimensional analyses in large cohorts of over 30 human tumors can be used to discover major cancer-causing genome alterations 28. TCGA portal (http://tumorsurvival.org/) is an interactive web-portal aimed at facilitating the TCGA-based data analysis. In our analysis, TCGA portal was used to draw survival curves to compare the overall survival of patients with higher and lower expressions of PSMB1-10. Clinicopathological features of 537 ccRCC patients and mRNA expression of PSMBs of 533 ccRCC patients were downloaded from the Firebrowse website (http://firebrowse.org/api-docs/). 4 of 537 ccRCC patients were excluded due to the absence of PSMBs mRNA expression data. Ultimately, 533 ccRCC patients with clinicopathological information and mRNA expression of PSMBs were included in our analysis. Clinical characteristics of the included patients, including gender, age, race, ethnicity, hemoglobin result, serum calcium, white cell count, pathologic stage, T, N, M, histologic grade were summarized in Table S1.

### cBioPortal

The cBioPortal (www.cbioportal.org) is an open-access resource for interactive exploration of multidimensional cancer genomics data sets, providing information regarding the integrative analysis of complex cancer genomics and clinical profiles from 105 cancer studies in the TCGA pipeline. The frequency of PSMB1-10 alterations (missense mutation, truncating mutation, amplification, deep deletion, mRNA high, mRNA low), copy number variance obtained from Genomic Identification of Significant Targets in Cancer, and mRNA expression z-scores were assessed using the cBioPortal for Cancer Genomics database and TCGA 29. Genetic mutations in PSMBs and their associations with OS of ccRCC patients were displayed as Kaplan-Meier plots and log-rank test was performed to identify the significance of the difference between the survival curves. When the p value <0.05, the difference was considered statistically significant.

### STRING

The Search Tool for Retrieval of Interacting Genes/Proteins (STRING) database is an interaction database that is dedicated to protein interactions at a wide scope, integrating both experimental interaction evidence and computational interaction prediction information, often including annotated pathway knowledge, text-mining results, inter-organism transfers or other accessory information 30, 31. In this study, we searched proteins that interact with PSMB1-10 using the “multiple proteins” channel, the minimum required interaction score was set as 0.4, with no more than 5 interactors shown in the first shell.

### GEPIA

Gene Expression Profiling Interactive Analysis (GEPIA) is an interactive web application for gene expression analysis based on RNA sequencing expression data of 9736 tumors and 8587 normal samples from the TCGA and the Genotype-Tissue Expression databases 32. GEPIA provides customizable functions such as tumor/normal differential analysis, survival analysis, similar gene detection, correlation analysis, and dimensionality reduction analysis. In this study, 50 similar genes of the gene set PSMB1-10 were identified using the GEPIA2 (http://gepia2.cancer-pku.cn) website.

### GO and KEGG analysis

Database for Annotation, Visualization, and Integrated Discovery (DAVID) (https://david.ncifcrf.gov/summary.jsp) bioinformatics resources consist of an integrated biological knowledgebase and analytic tools and provide functional interpretation of large lists of genes derived from genomic studies 33, 34. Gene ontology (GO) enrichment analysis, including biological processes (BP), cellular components (CC), and molecular functions (MF) were conducted for PSMB1-10 mutations and the other 50 selected genes to predict the functional roles of PSMBs mutations. Kyoto Encyclopedia of Genes and Genomes (KEGG) pathway analyses can define the pathways related to the PSMBs alterations and other selected genes associated with PSMBs mutations.

### Statistical methods

Bioinformatic statistics analyses were carried out using R v 4.1.1. Univariate and multivariate Cox regression analyses were applied to examine the prognostic value of PSMBs mRNA levels and clinicopathological parameters (including age, gender, histologic grade, pathologic stage, pathologic T, N, M).

## Results

### mRNA and protein expressions of PSMBs in patients with ccRCC

**Figure 1:**
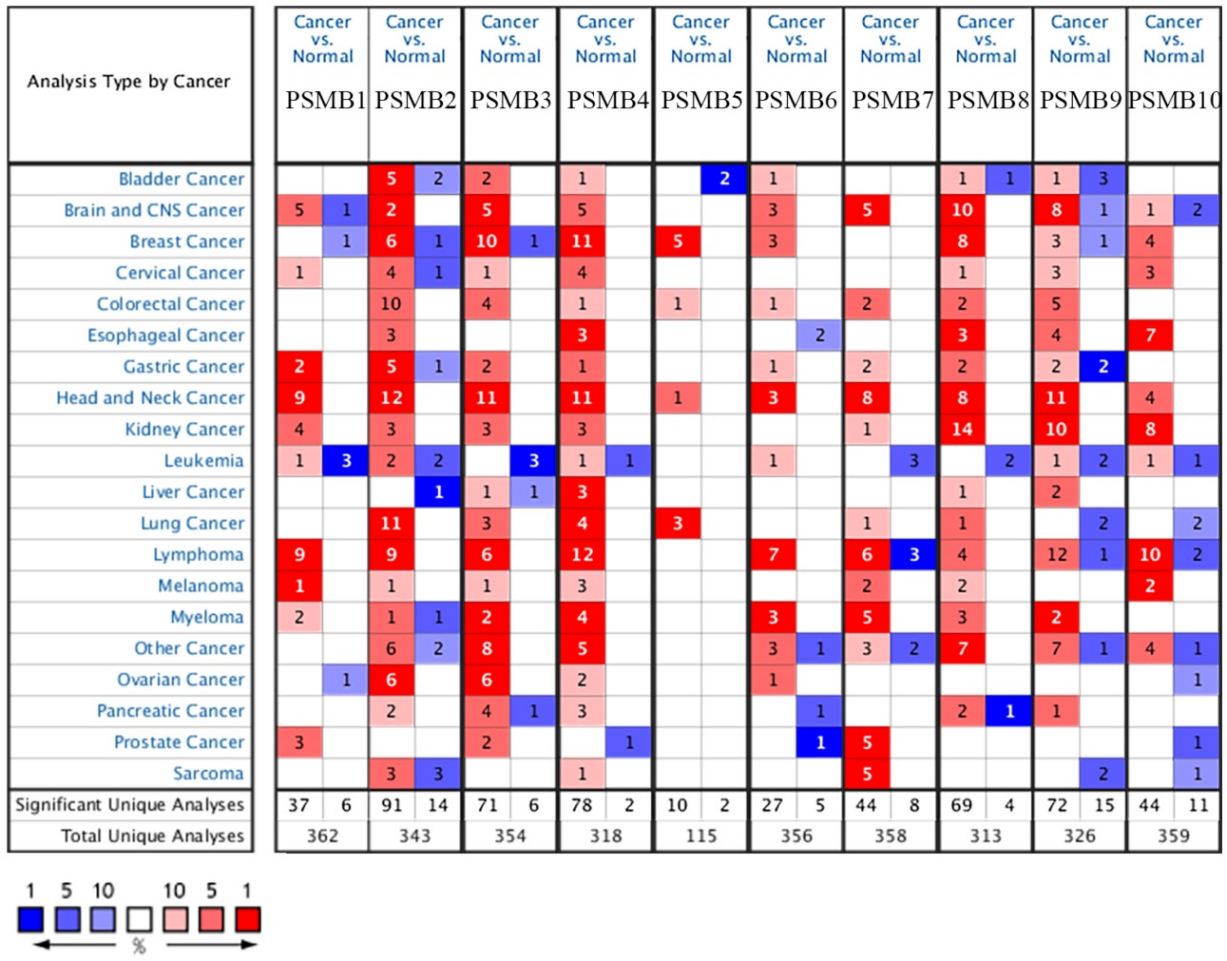
** Transcriptional expression of PSMB1-10 in 20 different types of cancers (ONCOMINE database).** Difference of transcriptional levels was compared by Student's t-test. Cut-off of p value and fold change: *p* value: 0.01, fold change: 1.5, data type: mRNA.

Transcriptional levels and protein expressions of PSMB1-10 in RCC and normal renal tissues were retrieved using the Oncomine, UALCAN, and HPA database. As were shown in Figure 1, transcriptional expressions of PSMB1-10 in 20 different types of cancers were compared to normal samples by Oncomine database. The mRNA levels of PSMB1-4 and PSMB7-10 were significantly upregulated in patients with kidney cancer in multiple datasets (Figure 1). Significant overexpression PSMB1, PSMB4, PSMB8, PSMB9 and PSMB10 in different types of RCC tissues were observed (Table 1). In Jones' dataset, PSMB1, PSMB4, PSMB9, PSMB10 were overexpressed in ccRCC versus normal renal tissue with a fold change of 1.763, 1.610, 5.267 and 2.595, respectively 35. In Lenburg's dataset, PSMB8, PSMB9, PSMB10 were overexpressed in ccRCC with a fold change of 2.980, 5.044 and 2.330, respectively 36. Similarly, in Yusenko's dataset, PSMB8, PSMB9, PSMB10 were overexpressed in ccRCC with a fold change of 2.980, 5.044 and 2.330, respectively 37. In Beroukhim's dataset, the mRNA levels of PSMB8, PSMB9 and PSMB10 were higher in non-hereditary ccRCC with a fold change of 4.678, 5.890 and 3.808, in hereditary ccRCC with a fold change of 5.235, 6.592 and 5.009, respectively, PSMB8 was overexpressed in ccRCC with a fold change of 15.139 38. In Gumz's dataset, PSMB9 and PSMB10 were overexpressed in ccRCC with a fold change of 4.688 and 2.829, respectively 39. In Higgins' dataset, PSMB8 was overexpressed in ccRCC versus normal renal tissue with a fold change of 2.765 40.

**Figure 2:**
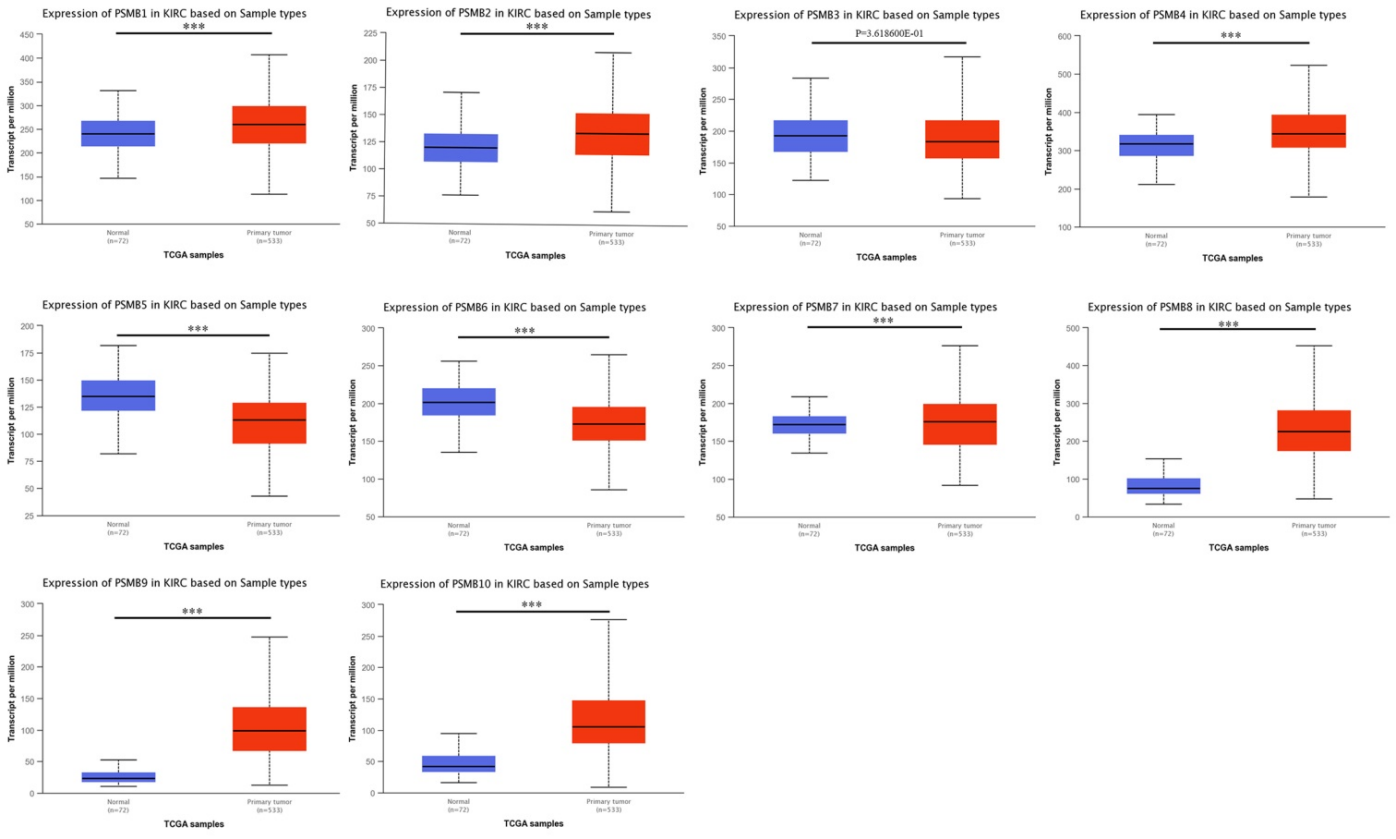
** mRNA expression of PSMBs in ccRCC tissues and adjacent normal renal tissues (UALCAN).** mRNA expressions of PSMB1, PSMB2, PSMB4, PSMB7-10 were found to be significantly elevated in RCC tissues compared to normal samples while mRNA expressions of PSMB5, PSMB6 were significantly reduced, no statistically differences of PSMB3 mRNA expressions were observed between cancer tissues and normal tissues. *** p<0.001.

We used UALCAN, an interactive web-portal performing in-depth analyses of TCGA gene expression data to measure the mRNA expression patterns of PSMB1-10 26. As was shown in Figure 2, mRNA expressions of PSMB1, PSMB2, PSMB4, PSMB7-10 were found to be significantly elevated in ccRCC tissues compared to normal samples while mRNA expressions of PSMB5, PSMB6 were significantly lower, no statistically difference being observed between cancer tissues and normal tissues with regards to PSMB3.

**Figure 3:**
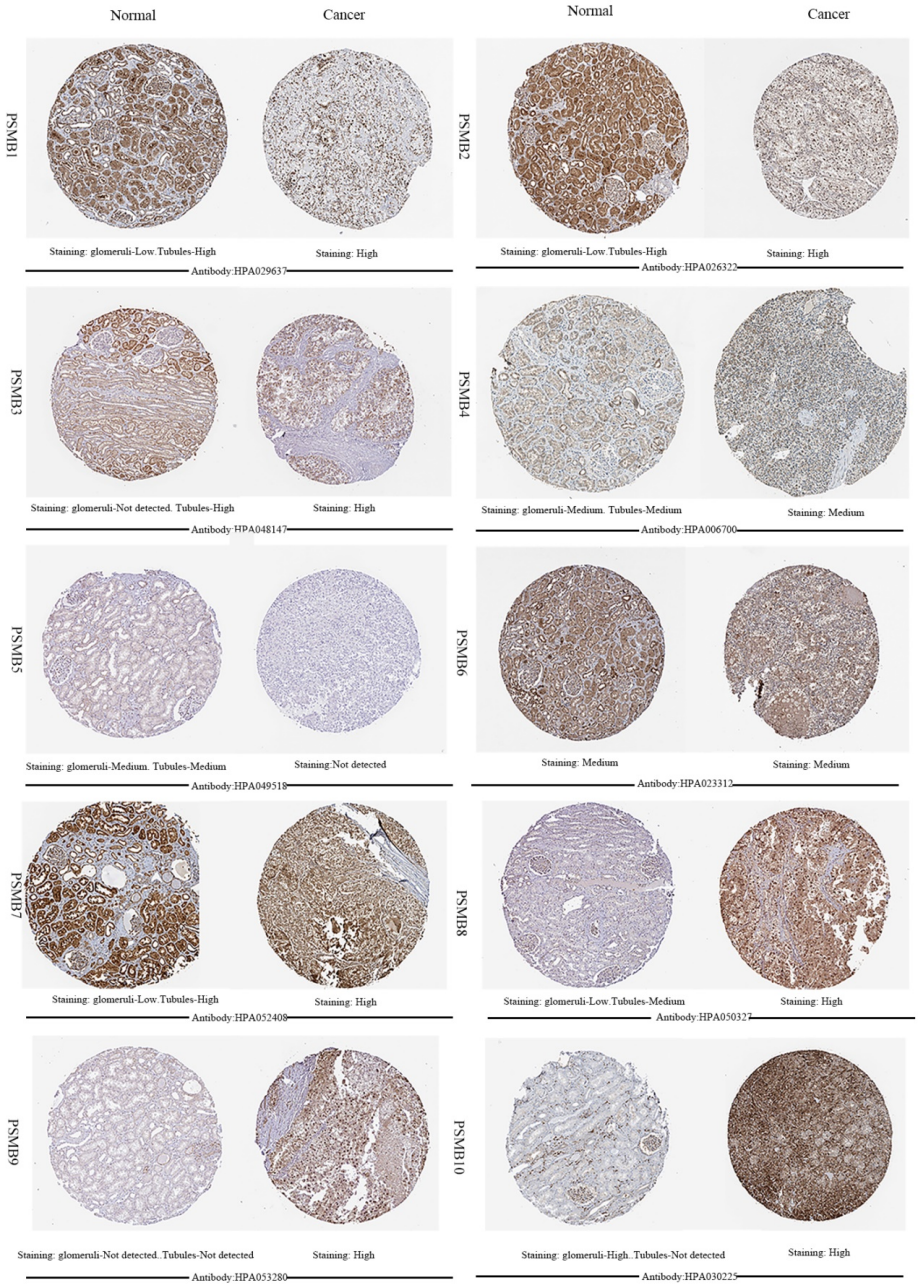
** Representative immunohistochemistry images of PSMB1-10 in ccRCC tissues and normal tissues (glomeruli and tubules) (Human Protein Atlas).** PSMB9 proteins were not detected in normal renal tissues, whereas their expressions were observed high in ccRCC tissues. Lower PSMB1/2/3/7/8 proteins expressions were observed in normal glomeruli compared with cancer tissues, lower PSMB8/10 proteins levels were observed in normal tubules compared with cancer tissues. PSMB5 proteins were detected staining medium in normal renal tissues and were not detected in cancer tissues. Staining of PSMB4/6 proteins was observed as medium in both normal tissues and cancer tissues.

Next, HPA was utilized to explore the expression levels of proteins encoded by PSMB1-10. As was shown in Figure 3, PSMB9 proteins were not detected in normal renal tissues, whereas their expressions were observed high in ccRCC tissues. Lower PSMB1/2/3/7/8 proteins expressions were observed in normal glomeruli compared with cancer tissues, and lower PSMB8/10 proteins levels were observed in normal tubules compared with cancer tissues. PSMB5 proteins were detected staining medium in normal renal tissues and were not detected in cancer tissues, which was consistent with the lower mRNA expression of PSMB5 observed in Figure 2. Staining of PSMB4/6 proteins was observed as medium in both normal tissues and cancer tissues.

**Figure 4:**
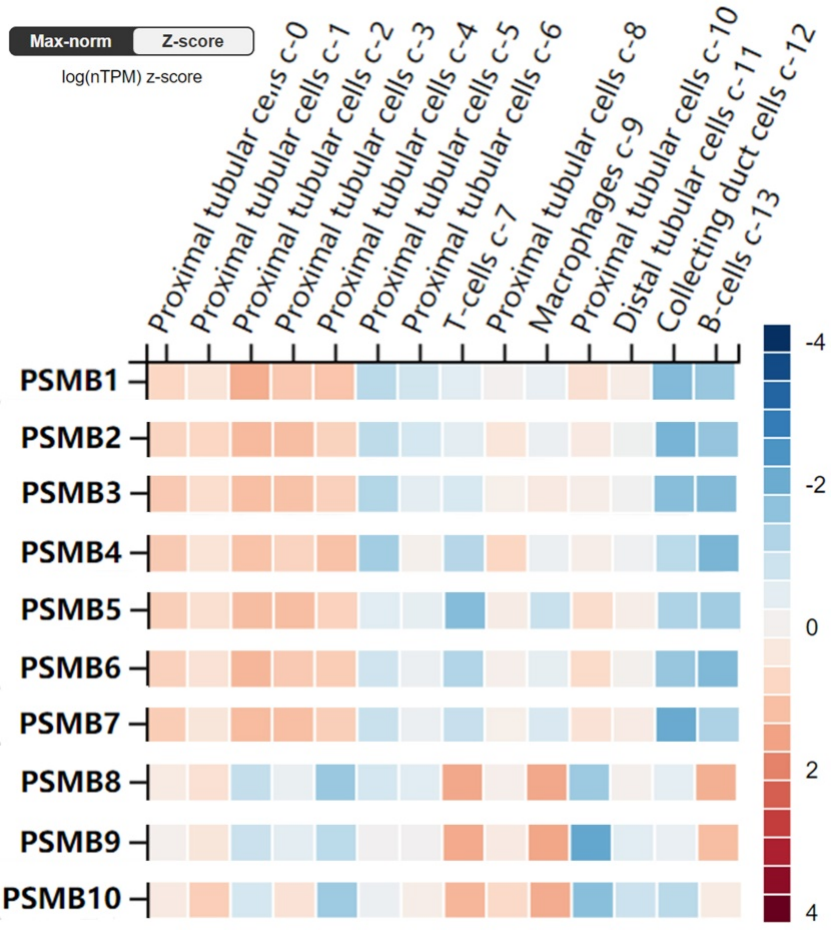
** RNA levels of PSMB1-10 in different single cell type clusters of the kidney (Human Protein Atlas)**. PSMB1-7 levels are higher in proximal tubular cells in kidney tissues, while PSMB8-10 are higher expressed in immune cells such as T cells, B cells and macrophages.

Furthermore, the RNA levels of PSMBs in healthy kidney tissues were investigated using the scRNA seq dataset of the HPA website. As was shown in Figure 4, PSMB1-7 mRNAs were significantly higher in proximal tubular cells, but were lower in B-cells and T-cells. By contrast, PSMB8-10 mRNAs were significantly higher in immune cells as B-cells, T-cells and macrophages, which might be explained by the fact that PSMB8-10 encode subunits of the immunoproteasome.

### Association of mRNA expression of PSMBs with pathological characteristics of ccRCC patients

**Figure 5:**
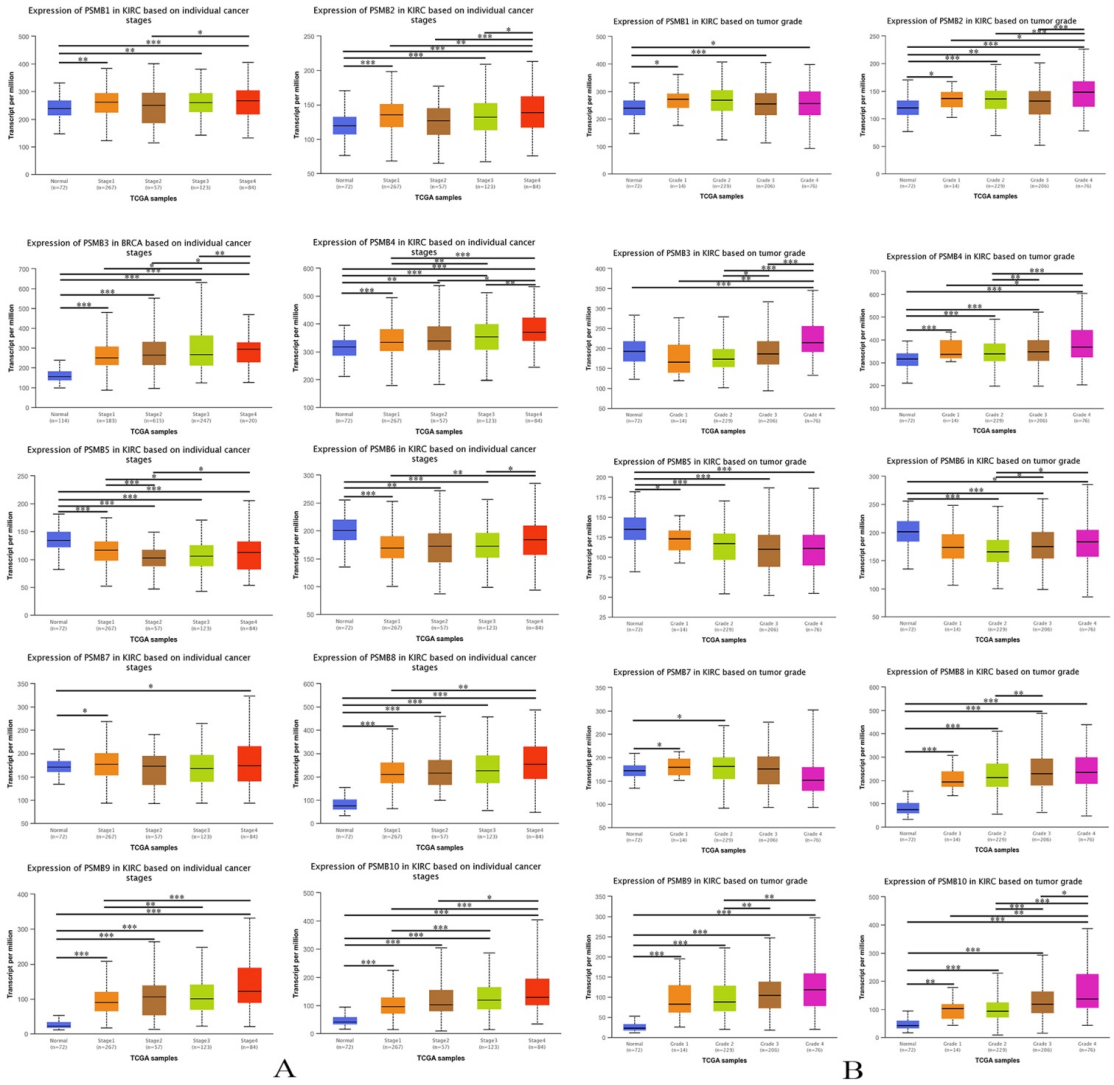
** Relationship between mRNA expression of PSMB1-10 and cancer stages and grades of ccRCC patients. A:** Higher mRNA levels of PSMB1/2/3/4/6/8/9/10 were associated with higher tumor stages significantly. **B:** Higher mRNA levels of PSMB2/3/4/6/8/9/10 were associated with higher tumor grades significantly. *p<0.05, **p<0.01, ***p<0.001.

To evaluate the association between mRNA levels of PSMBs and the pathological parameters of ccRCC patients, we conducted an analysis using UALCAN, tumor stage and histological grade chosen as two major parameters. As was shown in Figure 5A, mRNA expressions of PSMB1/2/3/4/6/8/9/10 were remarkably related to tumor stages, as the stage increased, an elevated tendency was observed in the mRNA expressions of PSMBs. Exceptionally, the mRNA expressions of PSMB1/2 in stage 2 were lower compared to those in stage 1; the mRNA levels of PSMB9 in stage 3 were lower than those in stage 2. Although the mRNA levels in PSMB6 saw a significant increase as tumor stage increased, the mRNA levels in tumors of either stage were lower than those expressed in normal renal tissues. From the figures, no significant correlations of PSMB7 mRNA levels and tumor stage were obtained, but a significant decrease in PSMB5 expression was found as the tumor stage increased.

As was shown in Figure 5B, mRNA levels of PSMB2/3/4/6/8/9/10 were remarkably related to tumor grade, as the grade progressed, the mRNA levels of PSMBs tended to be higher. However, the mRNA expressions of PSMB2 in grade 3 were lower compared to those in grade 1 and grade 2; similar to the lower mRNA expressions of PSMB10 in grade 2 in contrast to those in grade 1. Besides, mRNA levels of PSMB3 and PSMB6 in normal renal tissues were found to be higher than those in cancer tissues. Similar to the correlations of PSMBs and tumor stages in Figure 5A, the mRNA levels of PSMB1/7 did not increase while PSMB5 level decreased with the tumor grade.

### Prognostic value of mRNA PSMB1-10 in patients with ccRCC

**Figure 6:**
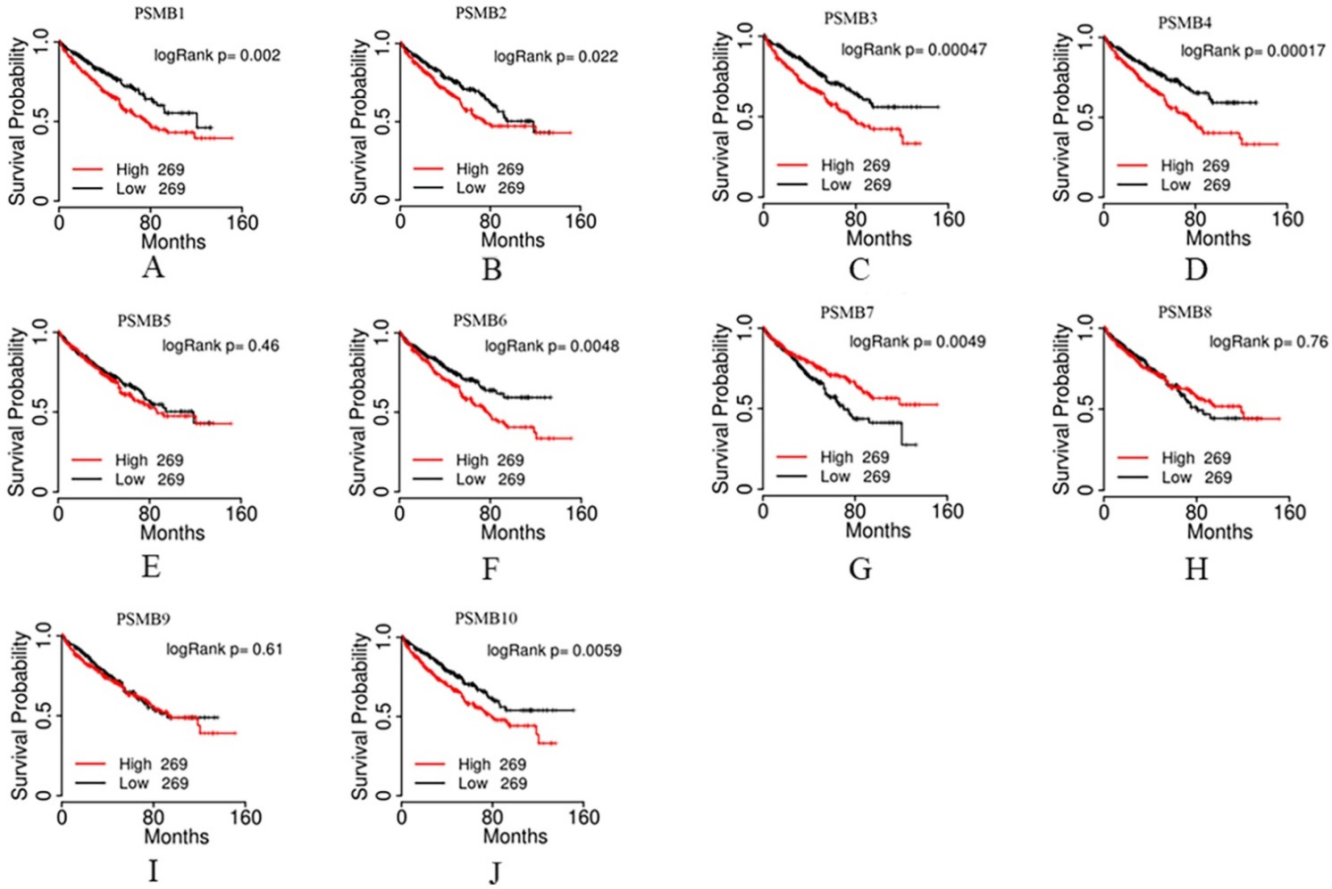
** Prognostic value of mRNA PSMB1-10 in patients with ccRCC.** Higher mRNA expressions of PSMB1/2/3/4/6/10 were significantly associated with shorter overall survival (OS). Higher mRNA expressions of PSMB7 were significantly associated with longer OS. No statistically significant correlations were observed between mRNA expressions of PSMB5/8/9 and OS of the patients.

We then evaluated the prognostic value of PSMBs in ccRCC by analyzing the associations of PSMB1-10 mRNA expression levels and overall survival (OS) of ccRCC patients using the TCGA portal. 538 cases were included in the analysis, the cases divided into two groups based on PSMB mRNA levels: the top half 269 cases with higher PSMB mRNA levels and the bottom half 269 cases with lower PSMB mRNA levels. As was shown in Figure 6, survival curves showed that higher mRNA levels of PSMB1/2/3/4/6/7/10 were significantly associated with shorter OS, whereas no statistically significant correlations were observed between mRNA expressions of PSMB5/8/9 and OS of the patients.

For an in-depth exploration of the correlations between PSMBs mRNA levels and patients' prognosis, we performed univariate and multivariate analysis based on data of the gene counts of PSMB1-10 of 533 ccRCC patients and corresponding clinicopathological parameters which were downloaded from TCGA database from the Firebrowse website. As was shown in Table S2, for the univariate analysis, age, histological grade, pathological stage, pathologic T, N, and M were positively associated with shorter OS with statistical significance. Higher transcriptional levels of PSMB1, 2, 3, 4, 6, 7 were found to be related to poorer OS. However, in the multivariate analysis, as was shown in Table S3-12, no significant correlations were observed between mRNA expressions of PSMB1-10 and OS of the 533 ccRCC patients.

### Genetic mutations in PSMBs and their associations with OS of ccRCC patients

**Figure 7:**
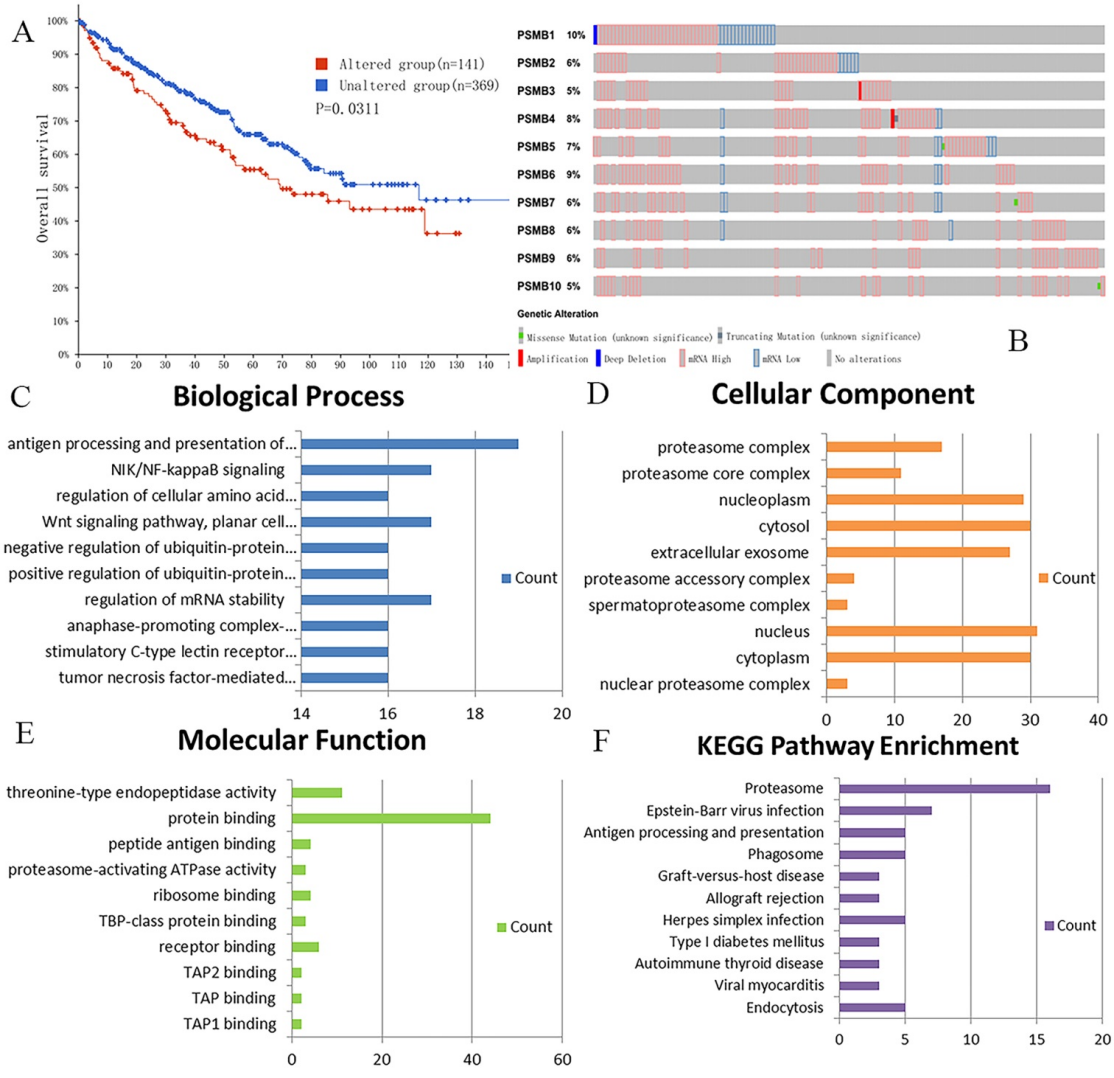
** Genetic mutations in PSMB1-10 and their association with OS of ccRCC patients (cBioPortal) and the enrichment analysis of mutations in PSMB1-10 and 50 similar genes (DAVID). A:** Genetic alterations in PSMBs were associated with shorter OS of ccRCC patients. **B:** PSMB1, PSMB6, PSMB4 and PSMB5 ranked the highest four genes of genetic alterations, and their mutation rates were 10%, 9%, 8% and 7%, respectively. **C:** GO biological process. **D:** GO cellular component. **E:** GO molecular function. **F:** Pathway enrichment based on KEGG.

We analyzed genetic alteration in PSMBs and their associations with OS of ccRCC patients using cBioPortal. As was shown in Figure 7A, high mutation rate of PSMBs was observed in ccRCC patients samples. Genetic alterations were found in 141 of the 510 sequenced ccRCC patients, accounting for a mutation rate of 28%. PSMB1, PSMB6, PSMB4 and PSMB5 ranked the highest four genes with genetic alterations, their mutation rates being 10%, 9%, 8% and 7%, respectively. Moreover, the Kaplan-Meier curve and log-rank test showed that genetic alterations in PSMBs were correlated with shorter OS (Figure 7B, p=0.0311) of ccRCC patients. These results indicated that genetic alterations of PSMBs could also have a significant influence on the prognosis of ccRCC patients.

### Exploration of PSMBs molecular functions and regulation pathways

**Figure 8:**
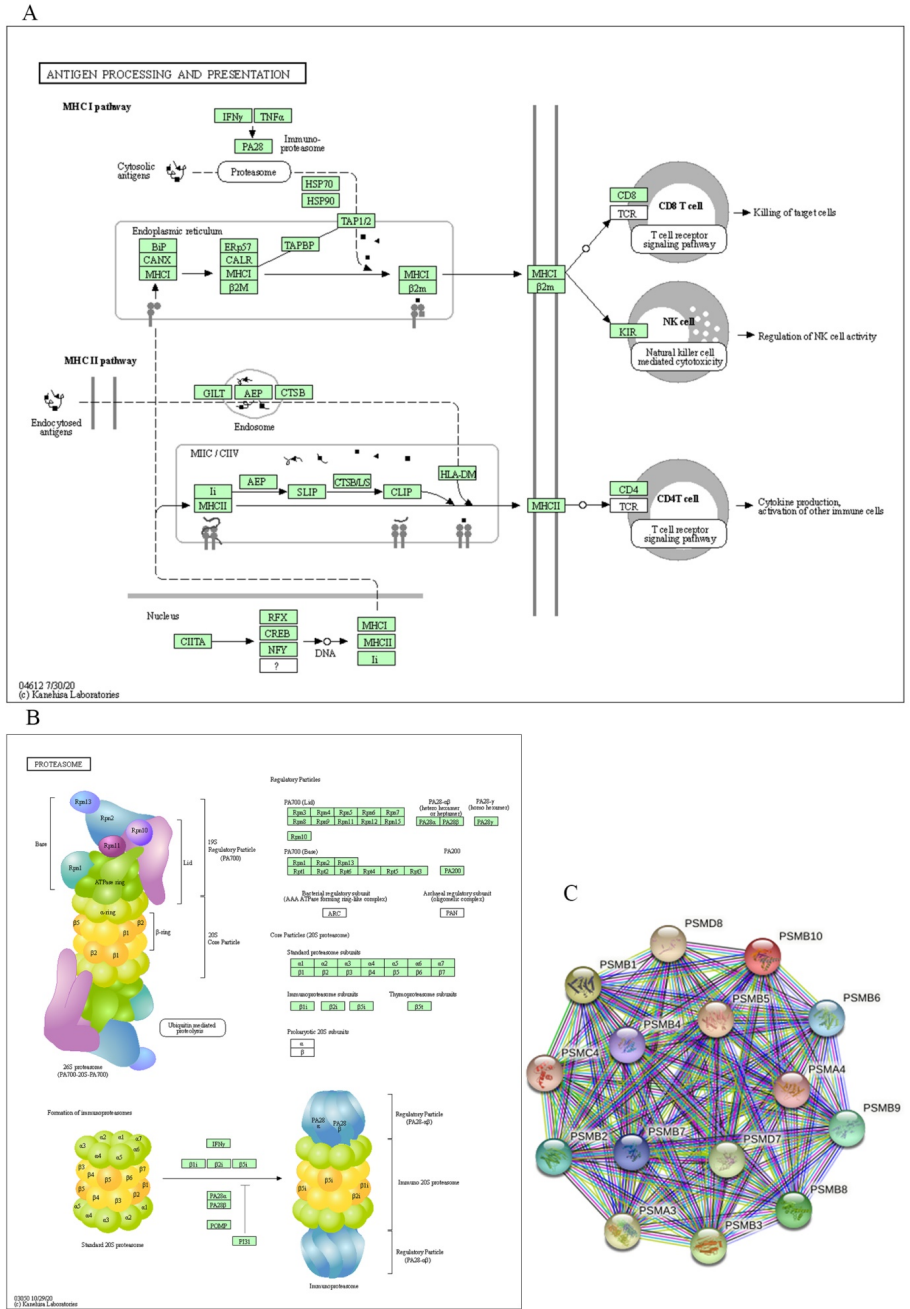
** Major molecular function and regulation pathway based on GO functional enrichment analysis and KEGG pathway analysis (DAVID) and the STRING interaction network of PSMB1-10 (STRING). A:** GO biological process: antigen processing and presentation. **B:** KEGG pathway: proteasome. **C:** STRING interaction network of PSMB1-10.

For functional enrichment analysis of PSMB family in ccRCC, we input PSMB1-10 as a gene set to detect similar genes using the expression analysis functions of GEPIA 2, obtaining top 50 genes (after getting rid of PSMB1-10) with highest Pearson correlation coefficient. Then, PSMB1-10 and the 50 similar genes were subjected to GO and KEGG analysis in DAVID. Figure 7C-F showed enriched pathways of BP, CC, MF and KEGG analysis, the top column of each chart represented pathway with the minimum p value. From the top column to the bottom column, the p value increases, all displaying pathways with p value<0.05. The length of the columns represented the number of genes enriched in that pathway. 73 GO enrichment items were statistically significant, the top 20 GO enrichment items being classified into three functional groups: BP group (17 items), MF group (1item), and CC group (2 items). The BP analysis suggested that these differentially expressed proteins were mainly involved in antigen processing and presentation of exogenous peptide antigen via major histocompatibility complex (MHC) class I, NIK/NF-κB (NF-κB inducing kinase/nuclear factor κ-light-chain-enhancer of activated B cells) signaling pathway, regulation of cellular amino acid metabolic process, WNT signaling pathway, and other related process. The MF analysis revealed that these differentially expressed proteins functioned mainly for threonine-type endopeptidase activity, protein binding, peptide antigen binding, and other related function. The CC analysis predicted cellular components regulated by these differentially expressed proteins, including proteasome complex, proteasome core complex, nucleoplasm, cytosol, etc. KEGG pathway analysis can define the molecular pathways in which PSMBs and other interacted genes were involved. The results showed 11 pathways that were related to the functions of PSMBs in ccRCC found through KEGG analysis (Figure 7F), including proteasome, Epstein-Barr virus infection, antigen processing and presentation, and phagosome. The top GO enrichment item “antigen processing and presentation of exogenous peptide antigen via MHC class I, TAP-dependent”, and the top KEGG enrichment pathway item “proteasome” were shown in Figure 8A, B.

Finally, we explored the STRING database to search for genes that interacted with PSMBs, as were shown in Figure 8C, PSMB1-10 were input to draw a protein-protein interaction network, no more than 5 clusters were set to show in the first shell. Ultimately, 15 proteins including PSMB1-10, PSMA3, PSMA4, PSMC4, PSMD7 and PSMD8 constructed the network. Active interaction sources included textmining, experiments, databases, co-expression, neighborhood, gene fusion and co-occurrence.

## Discussion

The proteasome, the main proteolysis machinery in human cells, plays a novel role in the regulation of cell cycle, cell survival and apoptosis, signal transduction, gene transcription and translation, and protein quality control 41. Emerging evidence reveals that the proteasome has a critical role in regulating proliferative signaling and anti-apoptotic pathways in many kinds of malignancies, including renal cancer 11, 41, 42. For instance, proteasomal degradation of two major tumor suppressors p53 and p27 has been observed in many types of cancers 10. The proteasome has been identified as a therapeutic target for a variety of malignancies. Bortezomib which strongly inhibit chymotrypsin-like and trypsin-like activity of the proteasome's β subunit was identified as an effective drug in malignancies such as multiple myeloma 17. However, the role of β subunit of proteasome, PSMB gene family, which is in charge of the major catalytic function of proteasome, in RCC has not been thoroughly unveiled. Notably, each member of PSMB gene family encodes subunit that occupies different position of the β-ring and possesses extremely different function 18. Yet, the distinct roles of specific PSMB family member played in the development and progression of RCC have not yet been completely elucidated either. In the present study, we have tried to assess the expressions of PSMB family members in RCC systematically by analyzing the mRNA, proteins in tumor tissue, correlation with tumor stage and grade, and overall survival. We also elaborated on the roles of distinct PSMB gene members in other malignancies. Finally, results from our study showed that higher mRNA expressions of PSMB1/2/4/7/8/9/10 were found in ccRCC tissues compared to normal tissues, transcriptional levels of PSMB2/3/4/6/8/9/10 were significantly positively related with patients' individual cancer stages and grades. Similar or higher levels of proteins encoded by PSMB1/2/3/7/8/9/10 were observed in tumor tissues compared to normal renal tissues. Higher levels of PSMB1/2/3/4/6/10 were significantly associated with shorter OS. The mutation of PSMB1, PSMB6, PSMB4 and PSMB5 ranked the four highest among the PSMBs family. While high levels of PSMB1/2/3/4/6/8/9/10 did not retain their prognostic significance in multivariate analysis, the consistent findings obtained through other analysis were predictive of their oncogenic activities.

PSMB1 encodes the subunit β6. This subunit has no known direct catalytic activity, but has been proposed to contribute to the assembly and structural stability of the proteasomes, enhancing the proteolytic environment on their inner surface 43. Ansar et al. reported that the incorporation of a small amount of the β6 mutant into proteasomes affected the chymotrypsin-like activity which was mediated by the β5 subunit of proteasome. Besides, the incorporation of impaired PSMB1 into 20S proteasomes resulted in lower proteasome amounts 44. Significant up-regulation of PSMB1 had been found in several malignancies, including lung adenocarcinoma, multiple myeloma and metastatic gastric cancer 21, 43, 45. Studies from Zhang et al. revealed that PSMC6 promoted cell growth and metastasis of lung adenocarcinoma by activating WNT signaling via degrading the AXIN, and PSMB1 and PSMB3 were positively correlated with PSMC6 in the gene set enrichment analysis 45. Varga et al. confirmed that patients with multiple myeloma carrying the variant allele of the PSMB1 P11A polymorphism had a significantly shorter progression-free survival 43. Similarly, subunits PSMB1 and PSMB6 were significantly enriched in serum exosomes derived from metastatic gastric cancer patients 21. In this study, higher mRNA expression of PSMB1 was found in ccRCC tissues compared to normal tissues, and was significantly related with patients' individual cancer stages. PSMB1 was also significantly related with shorter OS. The mutation of PSMB1 ranked the highest among the PSMBs family. All these results showed that PSMB1 played a role in the tumor development of ccRCC.

PSMB2 encodes the β4 subunit, a constitutive subunit which has no known direct proteolytic activity. PSMB2 overexpression is an oncogenic event in many kinds of malignancies, such as ovarian cancer, chronic myelogenous leukemia (CML), osteosarcoma, hepatocellular carcinoma (HCC) 22, 46-48. Wada et al. screened for genetic abnormalities by constructing retroviral expression libraries with the human ovarian cancer cell lines SHIN-3 and identified PSMB2 as ovarian cancer-related oncogenes 46. Similarly, Bruzzoni-Giovanelli et al. reported that single nucleotide polymorphisms (SNPs) identified in PSMB2 and PSMB10 were significantly associated with a predisposition to CML 22. Moreover, Zhou et al. had revealed that highly expressed PSMB2 was associated with poor osteosarcoma survival based on the bioinformatic analysis of four microarray data sets 47. Tan et al. showed that highly expressed PSMB2 predicted poorer prognosis of HCC, and that knockdown of PSMB2 suppressed HCC cell proliferation and invasion 48. In our study, significantly higher mRNA expression of PSMB2 was found in ccRCC tissues compared to normal tissues, and was positively related with patients' individual cancer stages and tumor grades. High PSMB2 expression was also correlated with poor OS in all and RCC patients, indicating its oncogenic role in renal cancer.

PSMB3 encodes the β3 subunit, a subunit which has no known direct proteolytic activity. In a human astrocytic cell line, siRNA-mediated knockdown of PSMB3 reduced proteasome expansions 49, 50. Overexpression of PSMB3 was also found to take part in tumor development 45, 51, 52. As previously mentioned, PSMB3 was involved in the cell growth and metastasis of lung adenocarcinoma 45. Moreover, Blijlevens et al. showed that PSMB3 overexpression promoted lung adenocarcinoma progression and corresponded to worse survival 51. Besides, PSMB3 has been reported to be positively correlated with ERBB2 in gene expression profiling of breast biopsies, the gene ERBB2 being an oncogene that was amplified in 10-40% of breast tumors 52. In the present study, no significant overexpression of PSMB3 mRNA was found in ccRCC tissues compared to normal tissues, but a positive correlation between the PSMB3 mRNA levels and patients' individual cancer stages and tumor grades was confirmed. Further, high PSMB3 expression was also correlated with worse OS in all RCC patients, indicating that PSMB3 took part in the tumorigenesis of RCC.

PSMB4, which encodes the β7 subunit, is the most frequently studied proteasomal subunit as well as the first identified subunit with oncogenic activities promoting tumor cell survival and tumor proliferation *in vivo* 53. During the assembly of β-ring, the β7 subunit is the last subunit incorporated in the precursor proteasomes 54 and has been found to play a key role in dimerization: its extended C-terminus being embedded in the channel between subunits β1 and β2 on the opposite ring, which facilitates a strong coupling of the two half-proteasomes 55, 56. Increased expression of the β7 subunit leads to a decrease in the level of precursor complex, indicating that the β7 subunit acts as rate-limiting subunit for their assembly 56. Deletion of the C-terminus of subunit β7 greatly decreased the efficiency of proteasome formation 55. For instance, significant decreases in protein levels of the three catalytic subunits β1, β2, and β5 were observed after knockdown of PSMB4 57. An increased level of PSMB4 has been observed in several solid cancers such as breast cancer, ovarian cancer, multiple myeloma, pulmonary neuroendocrine tumors and glioblastoma 58-63. A recent study revealed that PSMB4 overexpression in breast cancer cell lines and tissues enhanced the cell growth and viability and resulted in a poor prognosis 58. Mechanistically, Wang et al. proposed a PSMB4/NF-κB signaling pathway in breast cancer, suggesting that siRNA gene silencing of PSMB4 decreased NF-κB activity and cell viability, and caused cell cycle arrest at the G1/S phase 58. Cui et al. discovered that PSMB4 exhibited higher levels in both the tumors of transgenic mice and HCCs of human and functioned as an oncogene in HCC 62. Studies from Liu et al. showed that the mRNA level of PSMB4 was significantly associated with tumor grade, clinical stage, and lymphatic metastasis of epithelial ovarian cancer 60. Zhang et al. reported a significant promoting function of PSMB4 in multiple myeloma cell growth by activating NF-κB-miR-21 signaling 61. In this study, overexpression of PSMB4 mRNA and higher levels of proteins encoded by PSMB4 were found in ccRCC tissues compared to normal tissues, and was significantly related with patients' individual cancer stages and tumor grades. PSMB4 was also significantly related with shorter OS. The mutation of PSMB4 ranked the third highest among the PSMBs gene family. All these results showed that PSMB4 participated in the tumor development of ccRCC and could be identified as promising prognostic targets of RCC.

PSMB5 (also named X) encodes the β5 subunit, a subunit that contains the catalytic centers of chymotrypsin-like activity (hydrolyzes the peptide bond after large hydrophobic amino acid residues) of the proteasome 17. The PSMB5 has been reported to play a crucial role in facilitating the formation of functional proteasome and act as the step-limiting regulator in the process of proteasome-mediated protein degradation 64, 65. Overexpression and mutations of PSMB5 have been reported to contribute to drug resistance to proteasome inhibitors in several malignancies, such as multiple myeloma, breast cancer, and prostate cancer 66-68. In response to IFN-γ signaling, the three subunits PSMB5, PSMB6 and PSMB7 could be transformed into three very similar but different genes, PSMB8, PSMB9, and PSMB10, respectively, forming the so-called immunoproteasome. Overexpressions of PSMB5, PSMB6 and PSMB7 have been proposed to promote tumor development through inhibiting the activities of antigen-presenting MHC class I molecules which was partially performed by immunoproteasome 23, 69. As was reported by Wang et al., overexpression of PSMB5 suppressed the transformation of immune cells and promoted cell growth and migration of breast cancer. Furthermore, bioinformatics analysis revealed that up-regulation of PSMB5 was observed in breast cancer tissues and that overexpression of PSMB5 was predictive of worse survival 69. Beside, a recent study showed that PSMB5 was involved in the prostate cancer bone metastasis 70. However, the research conducted by Murakami et al. showed no significant correlation between the levels of PSMB5, PSMB6, PSMB7 and tumor grade, stage and survival of RCC 24. In the present study, no tumorigenic activities of PSMB5 were observed in RCC development. Down-regulated expression of PSMB5 mRNA and lower levels of proteins encoded by PSMB5 were found in ccRCC tissues compared to normal tissues, and no significant associations between PSMB5 transcriptional levels and patients' individual cancer stages and tumor grades and overall survival were observed. The mutation rate of PSMB5 ranked the fourth highest among the PSMBs gene family. All these results showed that PSMB5 might play a protective role in the tumor development of ccRCC, further researches are needed to add to the evidences and investigate the potential mechanisms. The functional role of PSMB5 in RCC awaits further experimental investigation.

PSMB6 (also named Y) encodes the β1 subunit, a subunit that contains the catalytic centers of caspase-like activity (hydrolyzes the peptide bond after negatively charged amino acid residues) of the proteasome 17. As was previously mentioned, the amounts of β1 which was encoded by PSMB6 and that of β1i which was encoded by PSMB9 were regulated by IFN-γ. At protein level, it was hypothesized that the regulation of PSMB6 and PSMB9 were likely to be reciprocal, that was, when PSMB6 was down-regulated, PSMB9 was up-regulated and vice versa. Therefore, overexpression of PSMB6 might promote tumor growth through immunosuppression induced by the insufficiency of PSMB9. In previous researches, PSMB6 was found to be up-regulated in metastatic gastric cancer but was not correlated with RCC development 21. In the present study, decreased expression of PSMB6 mRNA was found in ccRCC tissues compared to normal tissues, but a positive correlation between the PSMB6 mRNA levels and patients' individual cancer stages and tumor grades was confirmed. Moreover, high PSMB6 expression was also correlated with worse OS in all and RCC patients. Taken together, the oncogenic activity of PSMB6 in ccRCC was proposed despite the decreased PSMB6 mRNA expressions in ccRCC tissues compared with normal tissues. Further researches are needed to test the hypothesis.

PSMB7 encodes the β2 subunit, a subunit that contains the catalytic centers of trypsin-like activity (hydrolyzes the peptide bond mostly after positively charged amino acid residues) of the proteasome 17. PSMB7 also played an essential role in the assembly and structural stability of proteasome. Loss of β2 propeptide was reported to result in the failure of β3 recruitment and was therefore fatal 18. Similar to PSMB5, PSMB7 was found to contribute to anthracycline resistance and was predictive of significantly shorter survival in breast cancer 71. Furthermore, Rho et al. and Yoon et al. found that the level of PSB7 (the protein encoded by PSMB7) was increased in colorectal cancer tissues through proteomic expression analysis of surgical cancer tissues 23, 72. Whereas, similar to PSMB5 and PSMB6, no correlation between PSMB7 and RCC development was found in Murakami's research 24. In the present study, up-regulated PSMB7 mRNA was observed in ccRCC tissues compared to normal tissues. However, high PSMB7 expression was correlated with longer OS in all and RCC patients. No higher PSMB7 proteins were observed in RCC patients. Moreover, the results did not show a correlation between PSMB7 mRNA levels and the patients' individual cancer stages and tumor grades. All these results were insufficient to indicate a potential role of PSMB7 in the development of RCC. Therefore, further researches are still required to illustrate the exact role of PSMB7 in RCC.

PSMB8 (also known as LMP7) encodes the β5i subunit which has chymotrypsin-like activity. The stimulation of cells by IFN-γ activates the synthesis of three proteasomal subunits (β1i, β2i, and β5i), which during proteasome assembly are inserted instead of subunits β1, β2, and β5 17. The peptides generated by the immunoproteasome are not subjected to further degradation by proteolysis but are used for antigen presentation. The three immunoproteasomes PSMB8, PSMB9 and PSMB10 have been reported to play a dominant role in the surface display of peptide-MHC complexes. Low levels of the three subunits were established to cause a disorder in the antigen presentation system and thereby help the tumor cells to escape recognition and rejection by anti-tumor T cells in patients with RCC 24. Atkins et al. also showed down-regulations of PSMB8 and PSMB9 in RCC which were explained by reduced antigen presenting 73. Seliger et al. identified significant defects and down-regulations of PSMB8 and PSMB9 in RCC lesions, but the down-regulation was not associated with tumor grading 74. In Seliger's earlier research, down-regulated PSMB8 and PSMB9 were also observed in RCC cell lines and lymph node metastatic tissues compared to normal epithelial kidney cells 75. Whereas, Zhu et al. and Piotrowska et al. showed increased expression of the PSMB8 gene in RCC human tissues, with the ccRCC presenting highest PSMB8 levels among all histological types 76, 77. Besides, the aberrant expression of PSMB8 was observed in various malignancies, such as malignant melanoma, breast cancer, gastric cancer, esophageal squamous cell carcinoma, colorectal cancer and cervical cancer 72, 78-84. In our study, overexpression of PSMB8 mRNA and higher levels of proteins encoded by PSMB8 were found in ccRCC tissues compared to normal tissues, and was significantly related with patients' individual cancer stages and tumor grades. All these results showed that PSMB8 played a role in the development of RCC and could be identified as promising therapeutic targets of RCC.

PSMB9 (also known as LMP2) encodes the β1i subunit, another immunoproteasomal subunit which has caspase-like activity. As was previously mentioned, the low level of PSMB9 mRNA expressed in RCC and the possible mechanisms were similar to that of PSMB8. Van et al. constructed a mice model that harbored a disruption in PSMB9 gene and observed reduced antigen processing generated from CD8^+^ T lymphocytes 85. What was different from PSMB8 was that no up-regulation of PSMB9 was found in RCC in previous researches. Lack of PSMB9 was reported to trigger the malignant transformation from benign leiomyoma to uterine leiomyosarcoma 86-88. Previous studies that centered on the correlations between malignant tumors and PSMB9 mRNA expressions came to consistent conclusions. Lower PSMB9 mRNA level was observed in the late stage of malignant melanoma, breast cancer, esophageal carcinoma, pancreatic cancer and colon cancer 78, 79, 81, 83. In the present study, similar to PSMB8, up-regulated PSMB9 transcriptional levels and higher PSMB9 encoding protein levels were found in ccRCC tissues compared to normal tissues, and PSMB9 mRNA expression was significantly related with patients' individual cancer stages and tumor grades. To conclude, the results of our analysis showed that PSMB9 contributed to the development of ccRCC which conflicted with the lower expression of PSMB9 in RCC in published researches. Therefore, the exact role of PSMB9 in RCC was still unclear, further investigations of the underlying mechanisms are required.

PSMB10 (also known as LMP10) encodes the β2i subunit, one of the immunoproteasome catalytic subunits with trypsin-like activity. SNP of PSMB10 gene was found to increase the risk of CML, as was above mentioned 22. PSMB10 was also reported to be lower in metastatic breast cancer compared to primary breast cancer 79. Lower level of PSMB10 was shown to be strongly associated with shortened survival in RCC, loss of immune surveillance provided as an explanation 24. In our study, overexpression of PSMB10 mRNA and higher levels of proteins encoded by PSMB10 were found in ccRCC tissues compared to normal tissues, and was significantly related with patients' individual cancer stages and tumor grades. PSMB10 was also significantly related with shorter OS. Although published researches established a lower expression of PSMB10 in RCC, our analysis showed consistency in oncogenic activities of PSMB10 in RCC. Therefore, we believed that PSMB10 played a crucial role in the tumor development of RCC and could be identified as promising therapeutic targets of RCC.

## Supplementary Material

Supplementary tables.

## Figures and Tables

**Table 1:** Significant differences of PSMBs expression in transcription level between different types of RCC tissues and normal renal tissues (ONCOMINE)

Types of RCC vs. Kidney	Fold Change	P value	t-test	Ref
**PSMB1**				
ccRCC	1.763	5.15E-10	8.034	Jones Renal
**PSMB4**				
ccRCC	1.610	6.94E-09	7.150	Jones Renal
**PSMB8**				
ccRCC	2.980	2.37E-08	11.319	Lenburg Renal
ccRCC	2.765	2.92E-09	15.193	Higgins Renal
Non-Hereditary ccRCC	4.678	2.58E-13	13.127	Beroukhim Renal
Hereditary ccRCC	5.235	2.65E-12	16.332	Beroukhim Renal
ccRCC	15.139	1.08E-06	8.416	Beroukhim Renal
ccRCC	9.429	6.91E-05	11.121	Yusenko Renal
**PSMB9**				
ccRCC	5.044	3.11E-09	11.645	Lenburg Renal
ccRCC	4.688	1.88E-08	10.627	Gumz Renal
Non-Hereditary ccRCC	5.890	2.29E-09	9.496	Beroukhim Renal
Hereditary ccRCC	6.592	3.79E-09	11.072	Beroukhim Renal
ccRCC	5.267	1.70E-14	12.295	Jones Renal
ccRCC	12.722	7.25E-04	6.878	Yusenko Renal
**PSMB10**				
Non-Hereditary ccRCC	3.808	1.83E-09	8.547	Beroukhim Renal
Hereditary ccRCC	5.009	3.17E-10	12.130	Beroukhim Renal
ccRCC	6.000	5.74E-07	11.706	Yusenko Renal
ccRCC	2.330	4.24E-04	4.098	Lenburg Renal
ccRCC	2.595	5.49E-10	9.048	Jones Renal
ccRCC	2.829	6.21E-05	4.886	Gumz Renal

## Abbreviations

RCC: renal cell carcinoma
ccRCC: clear cell renal cell carcinoma
TCGA: The Cancer Genome Atlas
UPS: ubiquitin-proteasome system
pVHL: VHL protein
IFN: interferon
HPA: Human Protein Atlas
scRNA seq: single cell RNA sequencing
nTPM: normalized transcripts per million
STRING: Search Tool for Retrieval of Interacting Genes/Proteins
GEPIA: Gene Expression Profiling Interactive Analysis
DAVID: Database for Annotation, Visualization, and Integrated Discovery
GO: Gene Ontology
BP: biological process
CC: cellular component
MF: molecular function
KEGG: Kyoto Encyclopedia of Genes and Genomes
OS: overall survival
MHC: major histocompatibility complex
NF-κB: nuclear factor κ-light-chain-enhancer of activated B cells
NIK: NF-κB inducing kinase
CML: chronic myelogenous leukemia
HCC: hepatocellular carcinoma
SNP: single nucleotide polymorphism

## References

[1] Hsieh JJ, Purdue MP, Signoretti S, Swanton C, Albiges L, Schmidinger M (2017). Renal cell carcinoma. Nat Rev Dis Primers.

[2] Siegel RL, Miller KD, Jemal A (2018). Cancer statistics, 2018. CA Cancer J Clin.

[3] Linehan WM, Ricketts CJ (2019). The Cancer Genome Atlas of renal cell carcinoma: findings and clinical implications. Nat Rev Urol.

[4] Frank I, Blute ML, Cheville JC, Lohse CM, Weaver AL, Zincke H (2002). An outcome prediction model for patients with clear cell renal cell carcinoma treated with radical nephrectomy based on tumor stage, size, grade and necrosis: the SSIGN score. The Journal of urology.

[5] Patard JJ, Kim HL, Lam JS, Dorey FJ, Pantuck AJ, Zisman A (2004). Use of the University of California Los Angeles integrated staging system to predict survival in renal cell carcinoma: an international multicenter study. Journal of clinical oncology: official journal of the American Society of Clinical Oncology.

[6] Wolff I, May M, Hoschke B, Zigeuner R, Cindolo L, Hutterer G (2016). Do we need new high-risk criteria for surgically treated renal cancer patients to improve the outcome of future clinical trials in the adjuvant setting? Results of a comprehensive analysis based on the multicenter CORONA database. Eur J Surg Oncol.

[7] Meskawi M, Sun M, Trinh QD, Bianchi M, Hansen J, Tian Z (2012). A review of integrated staging systems for renal cell carcinoma. Eur Urol.

[8] Capitanio U, Bensalah K, Bex A, Boorjian SA, Bray F, Coleman J (2019). Epidemiology of Renal Cell Carcinoma. Eur Urol.

[9] Gerlinger M, Rowan AJ, Horswell S, Math M, Larkin J, Endesfelder D (2012). Intratumor heterogeneity and branched evolution revealed by multiregion sequencing. The New England journal of medicine.

[10] Shen M, Schmitt S, Buac D, Dou QP (2013). Targeting the ubiquitin-proteasome system for cancer therapy. Expert Opin Ther Targets.

[11] Mani A, Gelmann EP (2005). The ubiquitin-proteasome pathway and its role in cancer. Journal of clinical oncology: official journal of the American Society of Clinical Oncology.

[12] Corn PG (2007). Role of the ubiquitin proteasome system in renal cell carcinoma. BMC Biochem.

[13] An J, Sun Y, Fisher M, Rettig MB (2004). Maximal apoptosis of renal cell carcinoma by the proteasome inhibitor bortezomib is nuclear factor-kappaB dependent. Mol Cancer Ther.

[14] Kondagunta GV, Drucker B, Schwartz L, Bacik J, Marion S, Russo P (2004). Phase II trial of bortezomib for patients with advanced renal cell carcinoma. Journal of clinical oncology: official journal of the American Society of Clinical Oncology.

[15] Davis NB, Taber DA, Ansari RH, Ryan CW, George C, Vokes EE (2004). Phase II trial of PS-341 in patients with renal cell cancer: a University of Chicago phase II consortium study. Journal of clinical oncology: official journal of the American Society of Clinical Oncology.

[16] Li J, Pohl L, Schüler J, Korzeniewski N, Reimold P, Kaczorowski A (2021). Targeting the Proteasome in Advanced Renal Cell Carcinoma: Complexity and Limitations of Patient-Individualized Preclinical Drug Discovery. Biomedicines.

[17] Sorokin AV, Kim ER, Ovchinnikov LP (2009). Proteasome system of protein degradation and processing. Biochemistry (Mosc).

[18] Murata S, Yashiroda H, Tanaka K (2009). Molecular mechanisms of proteasome assembly. Nat Rev Mol Cell Biol.

[19] Miller Z, Ao L, Kim KB, Lee W (2013). Inhibitors of the immunoproteasome: current status and future directions. Current pharmaceutical design.

[20] Tomaru U, Ishizu A, Murata S, Miyatake Y, Suzuki S, Takahashi S (2009). Exclusive expression of proteasome subunit {beta}5t in the human thymic cortex. Blood.

[21] Ding XQ, Wang ZY, Xia D, Wang RX, Pan XR, Tong JH (2020). Proteomic Profiling of Serum Exosomes From Patients With Metastatic Gastric Cancer. Frontiers in oncology.

[22] Bruzzoni-Giovanelli H, González JR, Sigaux F, Villoutreix BO, Cayuela JM, Guilhot J (2015). Genetic polymorphisms associated with increased risk of developing chronic myelogenous leukemia. Oncotarget.

[23] Rho JH, Qin S, Wang JY, Roehrl MH (2008). Proteomic expression analysis of surgical human colorectal cancer tissues: up-regulation of PSB7, PRDX1, and SRP9 and hypoxic adaptation in cancer. Journal of proteome research.

[24] Murakami Y, Kanda K, Yokota K, Kanayama H, Kagawa S (2001). Prognostic significance of immuno-proteosome subunit expression in patients with renal-cell carcinoma: a preliminary study. Mol Urol.

[25] Rhodes DR, Yu J, Shanker K, Deshpande N, Varambally R, Ghosh D (2004). ONCOMINE: a cancer microarray database and integrated data-mining platform. Neoplasia.

[26] Chandrashekar DS, Bashel B, Balasubramanya SAH, Creighton CJ, Ponce-Rodriguez I, Chakravarthi B (2017). UALCAN: A Portal for Facilitating Tumor Subgroup Gene Expression and Survival Analyses. Neoplasia.

[27] Asplund A, Edqvist PH, Schwenk JM, Pontén F (2012). Antibodies for profiling the human proteome-The Human Protein Atlas as a resource for cancer research. Proteomics.

[28] Tomczak K, Czerwińska P, Wiznerowicz M (2015). The Cancer Genome Atlas (TCGA): an immeasurable source of knowledge. Contemp Oncol (Pozn).

[29] Cerami E, Gao J, Dogrusoz U, Gross BE, Sumer SO, Aksoy BA (2012). The cBio cancer genomics portal: an open platform for exploring multidimensional cancer genomics data. Cancer Discov.

[30] Szklarczyk D, Morris JH, Cook H, Kuhn M, Wyder S, Simonovic M (2017). The STRING database in 2017: quality-controlled protein-protein association networks, made broadly accessible. Nucleic Acids Res.

[31] Szklarczyk D, Gable AL, Lyon D, Junge A, Wyder S, Huerta-Cepas J (2019). STRING v11: protein-protein association networks with increased coverage, supporting functional discovery in genome-wide experimental datasets. Nucleic Acids Res.

[32] Tang Z, Li C, Kang B, Gao G, Li C, Zhang Z (2017). GEPIA: a web server for cancer and normal gene expression profiling and interactive analyses. Nucleic Acids Res.

[33] Huang DW, Sherman BT, Tan Q, Kir J, Liu D, Bryant D (2007). DAVID Bioinformatics Resources: expanded annotation database and novel algorithms to better extract biology from large gene lists. Nucleic Acids Res.

[34] Huang da W, Sherman BT, Lempicki RA (2009). Systematic and integrative analysis of large gene lists using DAVID bioinformatics resources. Nat Protoc.

[35] Jones J, Otu H, Spentzos D, Kolia S, Inan M, Beecken WD (2005). Gene signatures of progression and metastasis in renal cell cancer. Clinical cancer research: an official journal of the American Association for Cancer Research.

[36] Lenburg ME, Liou LS, Gerry NP, Frampton GM, Cohen HT, Christman MF (2003). Previously unidentified changes in renal cell carcinoma gene expression identified by parametric analysis of microarray data. BMC Cancer.

[37] Yusenko MV, Kuiper RP, Boethe T, Ljungberg B, van Kessel AG, Kovacs G (2009). High-resolution DNA copy number and gene expression analyses distinguish chromophobe renal cell carcinomas and renal oncocytomas. BMC Cancer.

[38] Beroukhim R, Brunet JP, Di Napoli A, Mertz KD, Seeley A, Pires MM (2009). Patterns of gene expression and copy-number alterations in von-hippel lindau disease-associated and sporadic clear cell carcinoma of the kidney. Cancer research.

[39] Gumz ML, Zou H, Kreinest PA, Childs AC, Belmonte LS, LeGrand SN (2007). Secreted frizzled-related protein 1 loss contributes to tumor phenotype of clear cell renal cell carcinoma. Clinical cancer research: an official journal of the American Association for Cancer Research.

[40] Higgins JP, Shinghal R, Gill H, Reese JH, Terris M, Cohen RJ (2003). Gene expression patterns in renal cell carcinoma assessed by complementary DNA microarray. Am J Pathol.

[41] Meyer-Schwesinger C (2019). The ubiquitin-proteasome system in kidney physiology and disease. Nat Rev Nephrol.

[42] Choi YH (2001). Proteasome-mediated degradation of BRCA1 protein in MCF-7 human breast cancer cells. International journal of oncology.

[43] Varga G, Mikala G, Kiss KP, Kosóczki É, Szabó E, Meggyesi N (2017). Proteasome Subunit Beta Type 1 P11A Polymorphism Is a New Prognostic Marker in Multiple Myeloma. Clin Lymphoma Myeloma Leuk.

[44] Ansar M, Ebstein F, Özkoç H, Paracha SA, Iwaszkiewicz J, Gesemann M (2020). Biallelic variants in PSMB1 encoding the proteasome subunit β6 cause impairment of proteasome function, microcephaly, intellectual disability, developmental delay and short stature. Hum Mol Genet.

[45] Zhang JY, Shi KZ, Liao XY, Li SJ, Bao D, Qian Y (2021). The Silence of PSMC6 Inhibits Cell Growth and Metastasis in Lung Adenocarcinoma. BioMed research international.

[46] Wada T, Yamashita Y, Saga Y, Takahashi K, Koinuma K, Choi YL (2009). Screening for genetic abnormalities involved in ovarian carcinogenesis using retroviral expression libraries. International journal of oncology.

[47] Zhou X, Fan Y, Ye W, Jia B, Yang Y, Liu Y (2020). Identification of the Novel Target Genes for Osteosarcoma Therapy Based on Comprehensive Bioinformatic Analysis. DNA Cell Biol.

[48] Tan S, Li H, Zhang W, Shao Y, Liu Y, Guan H (2018). NUDT21 negatively regulates PSMB2 and CXXC5 by alternative polyadenylation and contributes to hepatocellular carcinoma suppression. Oncogene.

[49] Concannon C, Lahue RS (2013). The 26S proteasome drives trinucleotide repeat expansions. Nucleic Acids Res.

[50] Jacquemont C, Taniguchi T (2007). Proteasome function is required for DNA damage response and fanconi anemia pathway activation. Cancer research.

[51] Blijlevens M, Komor MA, Sciarrillo R, Smit EF, Fijneman RJA, van Beusechem VW (2020). Silencing Core Spliceosome Sm Gene Expression Induces a Cytotoxic Splicing Switch in the Proteasome Subunit Beta 3 mRNA in Non-Small Cell Lung Cancer Cells. Int J Mol Sci.

[52] Dressman MA, Baras A, Malinowski R, Alvis LB, Kwon I, Walz TM (2003). Gene expression profiling detects gene amplification and differentiates tumor types in breast cancer. Cancer research.

[53] Lee GY, Haverty PM, Li L, Kljavin NM, Bourgon R, Lee J (2014). Comparative oncogenomics identifies PSMB4 and SHMT2 as potential cancer driver genes. Cancer research.

[54] Hirano Y, Kaneko T, Okamoto K, Bai M, Yashiroda H, Furuyama K (2008). Dissecting beta-ring assembly pathway of the mammalian 20S proteasome. Embo j.

[55] Ramos PC, Marques AJ, London MK, Dohmen RJ (2004). Role of C-terminal extensions of subunits beta2 and beta7 in assembly and activity of eukaryotic proteasomes. The Journal of biological chemistry.

[56] Marques AJ, Glanemann C, Ramos PC, Dohmen RJ (2007). The C-terminal extension of the beta7 subunit and activator complexes stabilize nascent 20 S proteasomes and promote their maturation. The Journal of biological chemistry.

[57] Valdagni R, Rancati T, Ghilotti M, Cozzarini C, Vavassori V, Fellin G (2009). To bleed or not to bleed. A prediction based on individual gene profiling combined with dose-volume histogram shapes in prostate cancer patients undergoing three-dimensional conformal radiation therapy. Int J Radiat Oncol Biol Phys.

[58] Wang H, He Z, Xia L, Zhang W, Xu L, Yue X (2018). PSMB4 overexpression enhances the cell growth and viability of breast cancer cells leading to a poor prognosis. Oncology reports.

[59] Zhang Y, Liu H, Cui M, Liu J, Yi R, Niu Y (2016). Effect of the HBV whole-X gene on the expression of hepatocellular carcinoma associated proteins. J Microbiol Immunol Infect.

[60] Liu R, Lu S, Deng Y, Yang S, He S, Cai J (2016). PSMB4 expression associates with epithelial ovarian cancer growth and poor prognosis. Arch Gynecol Obstet.

[61] Zheng P, Guo H, Li G, Han S, Luo F, Liu Y (2015). PSMB4 promotes multiple myeloma cell growth by activating NF-κB-miR-21 signaling. Biochemical and biophysical research communications.

[62] Cui F, Wang Y, Wang J, Wei K, Hu J, Liu F (2006). The up-regulation of proteasome subunits and lysosomal proteases in hepatocellular carcinomas of the HBx gene knockin transgenic mice. Proteomics.

[63] Mairinger FD, Walter RF, Theegarten D, Hager T, Vollbrecht C, Christoph DC (2014). Gene Expression Analysis of the 26S Proteasome Subunit PSMB4 Reveals Significant Upregulation, Different Expression and Association with Proliferation in Human Pulmonary Neuroendocrine Tumours. J Cancer.

[64] Chondrogianni N, Stratford FL, Trougakos IP, Friguet B, Rivett AJ, Gonos ES (2003). Central role of the proteasome in senescence and survival of human fibroblasts: induction of a senescence-like phenotype upon its inhibition and resistance to stress upon its activation. The Journal of biological chemistry.

[65] Chondrogianni N, Tzavelas C, Pemberton AJ, Nezis IP, Rivett AJ, Gonos ES (2005). Overexpression of proteasome beta5 assembled subunit increases the amount of proteasome and confers ameliorated response to oxidative stress and higher survival rates. The Journal of biological chemistry.

[66] Barrio S, Stühmer T, Da-Viá M, Barrio-Garcia C, Lehners N, Besse A (2019). Spectrum and functional validation of PSMB5 mutations in multiple myeloma. Leukemia.

[67] Wei W, Zou Y, Jiang Q, Zhou Z, Ding H, Yan L (2018). PSMB5 is associated with proliferation and drug resistance in triple-negative breast cancer. Int J Biol Markers.

[68] Singh V, Sharma V, Verma V, Pandey D, Yadav SK, Maikhuri JP (2015). Apigenin manipulates the ubiquitin-proteasome system to rescue estrogen receptor-β from degradation and induce apoptosis in prostate cancer cells. Eur J Nutr.

[69] Wang CY, Li CY, Hsu HP, Cho CY, Yen MC, Weng TY (2017). PSMB5 plays a dual role in cancer development and immunosuppression. Am J Cancer Res.

[70] Fan J, Du W, Zhang H, Wang Y, Li K, Meng Y (2020). Transcriptional downregulation of miR-127-3p by CTCF promotes prostate cancer bone metastasis by targeting PSMB5. FEBS Lett.

[71] Munkácsy G, Abdul-Ghani R, Mihály Z, Tegze B, Tchernitsa O, Surowiak P (2010). PSMB7 is associated with anthracycline resistance and is a prognostic biomarker in breast cancer. British journal of cancer.

[72] Yoon JY, Wang JY, Roehrl MHA (2020). An Investigation Into the Prognostic Significance of High Proteasome PSB7 Protein Expression in Colorectal Cancer. Front Med (Lausanne).

[73] Atkins D, Ferrone S, Schmahl GE, Störkel S, Seliger B (2004). Down-regulation of HLA class I antigen processing molecules: an immune escape mechanism of renal cell carcinoma?. The Journal of urology.

[74] Seliger B, Atkins D, Bock M, Ritz U, Ferrone S, Huber C (2003). Characterization of human lymphocyte antigen class I antigen-processing machinery defects in renal cell carcinoma lesions with special emphasis on transporter-associated with antigen-processing down-regulation. Clinical cancer research: an official journal of the American Association for Cancer Research.

[75] Seliger B, Höhne A, Knuth A, Bernhard H, Ehring B, Tampé R (1996). Reduced membrane major histocompatibility complex class I density and stability in a subset of human renal cell carcinomas with low TAP and LMP expression. Clinical cancer research: an official journal of the American Association for Cancer Research.

[76] Zhu S, Huang Y, Su X (2016). Mir-451 Correlates with Prognosis of Renal Cell Carcinoma Patients and Inhibits Cellular Proliferation of Renal Cell Carcinoma. Medical science monitor: international medical journal of experimental and clinical research.

[77] Piotrowska Ż, Niezgoda M, Młynarczyk G, Acewicz M, Kasacka I (2020). Comparative Assessment of the WNT/β-Catenin Pathway, CacyBP/SIP, and the Immunoproteasome Subunit LMP7 in Various Histological Types of Renal Cell Carcinoma. Frontiers in oncology.

[78] Dissemond J, Goette P, Moers J, Lindeke A, Goos M, Ferrone S (2003). Immunoproteasome subunits LMP2 and LMP7 downregulation in primary malignant melanoma lesions: association with lack of spontaneous regression. Melanoma Res.

[79] Szekely B, Bossuyt V, Li X, Wali VB, Patwardhan GA, Frederick C (2018). Immunological differences between primary and metastatic breast cancer. Annals of oncology: official journal of the European Society for Medical Oncology.

[80] Kwon CH, Park HJ, Choi YR, Kim A, Kim HW, Choi JH (2016). PSMB8 and PBK as potential gastric cancer subtype-specific biomarkers associated with prognosis. Oncotarget.

[81] Liu Q, Hao C, Su P, Shi J (2009). Down-regulation of HLA class I antigen-processing machinery components in esophageal squamous cell carcinomas: association with disease progression. Scand J Gastroenterol.

[82] Kang JK, Yoon SJ, Kim NK, Heo DS (2000). The expression of MHC class I, TAP1/2, and LMP2/7 gene in human gastric cancer cell lines. International journal of oncology.

[83] Imanishi T, Kamigaki T, Nakamura T, Hayashi S, Yasuda T, Kawasaki K (2006). Correlation between expression of major histocompatibility complex class I and that of antigen presenting machineries in carcinoma cell lines of the pancreas, biliary tract and colon. Kobe J Med Sci.

[84] Li C, Dai S, Yan Z, Zhang X, Liu S, Wang X (2020). Genetic polymorphisms of proteasome subunit genes of the MHC-I antigen-presenting system are associated with cervical cancer in a Chinese Han population. Hum Immunol.

[85] Van Kaer L, Ashton-Rickardt PG, Eichelberger M, Gaczynska M, Nagashima K, Rock KL (1994). Altered peptidase and viral-specific T cell response in LMP2 mutant mice. Immunity.

[86] Hayashi T, Horiuchi A, Sano K, Hiraoka N, Kanai Y, Shiozawa T (2010). Mice-lacking LMP2, immuno-proteasome subunit, as an animal model of spontaneous uterine leiomyosarcoma. Protein Cell.

[87] Hayashi T, Horiuchi A, Sano K, Hiraoka N, Ichimura T, Sudo T (2014). Potential diagnostic biomarkers: differential expression of LMP2/β1i and cyclin B1 in human uterine leiomyosarcoma. Tumori.

[88] Hayashi T, Kawano M, Sano K, Ichimura T, Gur G, Yaish P (2017). A novel diagnostic biomarker for human uterine leiomyosarcoma: PSMB9/β1i. Chin Clin Oncol.

