# Supplementary material for: Bioinformatic Analysis Identifying PSMB 1/2/3/4/6/8/9/10 as Prognostic Indicators in Clear Cell Renal Cell Carcinoma
Source: Int J Med Sci. 2022 May 1;19(5):796–812. doi: 10.7150/ijms.71152 (PMC9149646; doi:10.7150/ijms.71152)
Supplement: Supplementary file 1 — Supplementary tables. [file ijmsv19p0796s1.pdf]

Table S1. Basic characteristics of 533 ccRCC patients.

**Variables**	**ccRCC patients (N=533)**
**Gender (Male/female)**	345/188
**Age (years, Mean ± SD)**	60.63±12.14
**Race**	
Asian	N=8
White	N=462
Black or African American	N=56
**Ethnicity**	
Hispanic or latino	N=26
Not hispanic or latino	N=355
**Hemoglobin result**	
Elevated	N=5
Normal	N=185
Low	N=262
**Serum calcium**	
Elevated	N=10
Normal	N=150
Low	N=204
**White cell count**	
Elevated	N=164
Normal	N=267
Low	N=8
**Pathologic stage**	
Stage I	N=267
Stage II	N=57
Stage III	N=123
Stage IV	N=84
**Histologic grade**	
Grade1	N=14
Grade2	N=229
Grade3	N=206
Grade4	N=76
**Pathologic T**	
T1	N=273
T2	N=69
T3	N=180
T4	N=11
**Pathologic N**	
N0	N=240
N1	N=16
**Pathologic M**	
M0	N=422
M1	N=79

Table S2. Univariate analysis of overall survival in 533 ccRCC specimens.

Variables	Univariate analysis		
	**Hazard ratio**	**95% CI**	**P value**
Age (years)	1.033	1.019-1.047	<0.001*
Gender	0.931	0.675-1.284	0.663
Histologic grade	2.293	1.854-2.836	<0.001*
Pathologic stage	1.889	1.649-2.164	<0.001*
Pathologic T	1.941	1.639-2.299	<0.001*
Pathologic N	0.996	0.993-0.999	0.020*
Pathologic M	4.284	3.106-5.908	<0.001*
PSMB1	1.520	1.036-2.231	0.032*
PSMB2	2.387	1.437-3.965	0.001*
PSMB3	1.786	1.394-2.289	<0.001*
PSMB4	2.083	1.434-3.026	<0.001*
PSMB5	1.328	0.884-1.993	0.171
PSMB6	1.674	1.196-2.342	0.003*
PSMB7	0.569	0.363-0.890	0.014*
PSMB8	1.025	0.778-1.351	0.860
PSMB9	1.078	0.884-1.314	0.460
PSMB10	1.233	0.994-1.529	0.057

Table S3. Multivariate analysis of overall survival in 533 ccRCC specimens.

Variables	Multivariate analysis		
	**Hazard ratio**	**95% CI**	**P value**
Age (years)	1.035	1.020-1.051	<0.001*
Gender	1.019	0.732-1.420	0.909
Histologic grade	1.575	1.238-2.002	<0.001*
Pathologic stage	1.598	1.022-2.499	0.040*
Pathologic T	0.906	0.603-1.361	0.633
Pathologic N	0.996	0.993-0.999	0.018*
Pathologic M	1.411	0.718-2.774	0.318
PSMB1	1.002	0.992-1.011	0.701

Table S4. Multivariate analysis of overall survival in 533 ccRCC specimens.

Variables	Multivariate analysis		
	Hazard ratio	95% CI	P value
Age (years)	1.036	1.020-1.051	<0.001*
Gender	1.032	0.740-1.440	0.853
Histologic grade	1.547	1.210-1.980	0.001*
Pathologic stage	1.590	1.018-2.484	0.042*
Pathologic T	0.907	0.604-1.361	0.637
Pathologic N	0.996	0.993-0.999	0.016*
Pathologic M	1.409	0.719-2.763	0.318
PSMB2	1.011	0.971-1.051	0.604

Table S5. Multivariate analysis of overall survival in 533 ccRCC specimens.

Variables	Multivariate analysis		
	Hazard ratio	95% CI	P value
Age (years)	1.035	1.020-1.051	<0.001*
Gender	1.021	0.733-1.422	0.904
Histologic grade	1.549	1.217-1.971	<0.001*
Pathologic stage	1.584	1.016-2.469	0.042*
Pathologic T	0.912	0.609-1.366	0.655
Pathologic N	0.996	0.993-0.999	0.018*
Pathologic M	1.425	0.727-2.794	0.303
PSMB3	1.002	0.998-1.007	0.332

Table S6. Multivariate analysis of overall survival in 533 ccRCC specimens.

Variables	Multivariate analysis		
	Hazard ratio	95% CI	P value
Age (years)	1.035	1.020-1.051	<0.001*
Gender	1.015	0.729-1.415	0.928
Histologic grade	1.557	1.224-1.981	<0.001*
Pathologic stage	1.561	0.994-2.451	0.053
Pathologic T	0.922	0.613-1.386	0.695
Pathologic N	0.996	0.993-0.999	0.015*
Pathologic M	1.443	0.733-2.841	0.289
PSMB4	1.002	0.996-1.008	0.453

Table S7. Multivariate analysis of overall survival in 533 ccRCC specimens.

Variables	Multivariate analysis		
	**Hazard ratio**	**95% CI**	**P value**
Age (years)	1.036	1.020-1.051	<0.001*
Gender	0.987	0.707-1.378	0.938
Histologic grade	1.571	1.238-1.994	<0.001*
Pathologic stage	1.714	1.101-2.669	0.017*
Pathologic T	0.845	0.563-1.268	0.416
Pathologic N	0.996	0.993-0.999	0.014*
Pathologic M	1.292	0.660-2.531	0.455
PSMB5	1.009	0.998-1.020	0.111

Table S8. Multivariate analysis of overall survival in 533 ccRCC specimens.

Variables	Multivariate analysis		
	**Hazard ratio**	**95% CI**	**P value**
Age (years)	1.035	1.020-1.051	<0.001*
Gender	1.018	0.730-1.418	0.917
Histologic grade	1.561	1.226-1.986	<0.001*
Pathologic stage	1.593	1.020-2.488	0.041*
Pathologic T	0.910	0.606-1.366	0.648
Pathologic N	0.996	0.993-0.999	0.017*
Pathologic M	1.417	0.722-2.782	0.311
PSMB6	1.002	0.994-1.010	0.613

Table S9. Multivariate analysis of overall survival in 533 ccRCC specimens.

Variables	Multivariate analysis		
	**Hazard ratio**	**95% CI**	**P value**
Age (years)	1.035	1.020-1.051	<0.001*
Gender	1.019	0.734-1.428	0.892
Histologic grade	1.575	1.232-2.010	<0.001*
Pathologic stage	1.598	1.029-2.513	0.037*
Pathologic T	0.906	0.601-1.359	0.627
Pathologic N	0.996	0.993-0.999	0.016*
Pathologic M	1.411	0.715-2.764	0.324
PSMB7	1.002	0.990-1.011	0.950

Table S10. Multivariate analysis of overall survival in 533 ccRCC specimens.

**Variables**	**Multivariate analysis**		
	**Hazard ratio**	**95% CI**	**P value**
Age (years)	1.035	1.020-1.051	<0.001*
Gender	0.979	0.701-1.367	0.900
Histologic grade	1.616	1.270-2.056	<0.001*
Pathologic stage	1.742	1.101-2.757	0.018*
Pathologic T	0.840	0.551-1.281	0.418
Pathologic N	0.996	0.993-0.999	0.011*
Pathologic M	1.318	0.668-2.601	0.426
PSMB8	0.995	0.990-1.001	0.090

Table S11. Multivariate analysis of overall survival in 533 ccRCC specimens.

**Variables**	**Multivariate analysis**		
	**Hazard ratio**	**95% CI**	**P value**
Age (years)	1.035	1.020-1.051	<0.001*
Gender	0.995	0.713-1.390	0.978
Histologic grade	1.604	1.260-2.041	<0.001*
Pathologic stage	1.665	1.054-2.630	0.029*
Pathologic T	0.880	0.579-1.340	0.552
Pathologic N	0.996	0.993-0.999	0.012*
Pathologic M	1.376	0.696-2.720	0.359
PSMB9	0.993	0.983-1.004	0.225

Table S12. Multivariate analysis of overall survival in 533 ccRCC specimens.

**Variables**	**Multivariate analysis**		
	**Hazard ratio**	**95% CI**	**P value**
Age (years)	1.036	1.020-1.051	<0.001*
Gender	1.014	0.728-1.413	0.933
Histologic grade	1.596	1.252-2.035	<0.001*
Pathologic stage	1.662	1.054-2.622	0.029*
Pathologic T	0.884	0.585-1.338	0.561
Pathologic N	0.996	0.993-0.999	0.015*
Pathologic M	1.358	0.687-2.683	0.379
PSMB10	0.994	0.980-1.009	0.448
